# 4-(2-{[4-Amino-6-(4-nitro­benz­yl)-5-oxo-4,5-dihydro-1,2,4-triazin-3-yl]sulfan­yl}acet­yl)-3-phenyl­sydnone

**DOI:** 10.1107/S1600536811014504

**Published:** 2011-04-22

**Authors:** Hoong-Kun Fun, Mohd Mustaqim Rosli, Nithinchandra, Balakrishna Kalluraya

**Affiliations:** a X-ray Crystallography Unit, School of Physics, Universiti Sains Malaysia, 11800 USM, Penang, Malaysia; b Department of Studies in Chemistry, Mangalore University, Mangalagangotri, Mangalore 574 199, India

## Abstract

In the crystal, C_20_H_15_N_7_O_6_S, the dihedral angle between the oxadiazole and triazine rings is 86.94 (7)°. The oxadiazole ring makes a dihedral angle of 52.96 (8)° with the phenyl ring, while the triazine ring makes a dihedral angle of 82.08 (7)° with the benzene ring. In the structure, mol­ecules are linked by a pair of N—H⋯O hydrogen bonds, forming an inversion dimer. The dimers are further stacked along the *a* axis *via* N—H⋯N hydrogen bonds. Weak inter­molecular C—H⋯O inter­actions are also observed.

## Related literature

For the biological activity of sydnone derivatives, see: Rai *et al.* (2008 ▶); Jyothi *et al.* (2008 ▶); Kalluraya *et al.* (2008*a* ▶,*b* ▶). For a related structure, see: Fun *et al.* (2011 ▶).
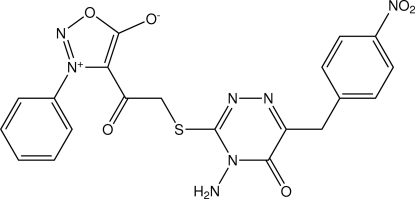


## Experimental

### 

#### Crystal data

C_20_H_15_N_7_O_6_S
*M* *_r_* = 481.45
Triclinic, 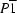

*a* = 6.4071 (1) Å
*b* = 10.1629 (2) Å
*c* = 17.1521 (3) Å
α = 106.372 (1)°
β = 92.400 (1)°
γ = 97.551 (1)°
*V* = 1058.61 (3) Å^3^
*Z* = 2
Mo *K*α radiation
μ = 0.21 mm^−1^
*T* = 297 K
0.51 × 0.34 × 0.17 mm

#### Data collection

Bruker SMART APEXII CCD area-detector diffractometer
Absorption correction: multi-scan (*SADABS*; Bruker, 2009 ▶) *T*_min_ = 0.900, *T*_max_ = 0.965
21821 measured reflections
7793 independent reflections
5330 reflections with *I* > 2σ(*I*)
*R*_int_ = 0.030

#### Refinement

*R*[*F* ^2^ > 2σ(*F* ^2^)] = 0.047
*wR*(*F* ^2^) = 0.131
*S* = 1.04
7793 reflections
315 parameters
H atoms treated by a mixture of independent and constrained refinement
Δρ_max_ = 0.25 e Å^−3^
Δρ_min_ = −0.23 e Å^−3^

### 

Data collection: *APEX2* (Bruker, 2009 ▶); cell refinement: *SAINT* (Bruker, 2009 ▶); data reduction: *SAINT*; program(s) used to solve structure: *SHELXTL* (Sheldrick, 2008 ▶); program(s) used to refine structure: *SHELXTL*; molecular graphics: *SHELXTL*; software used to prepare material for publication: *SHELXTL* and *PLATON* (Spek, 2009 ▶).

## Supplementary Material

Crystal structure: contains datablocks global, I. DOI: 10.1107/S1600536811014504/is2702sup1.cif

Structure factors: contains datablocks I. DOI: 10.1107/S1600536811014504/is2702Isup2.hkl

Additional supplementary materials:  crystallographic information; 3D view; checkCIF report

## Figures and Tables

**Table 1:** Hydrogen-bond geometry (Å, °)

*D*—H⋯*A*	*D*—H	H⋯*A*	*D*⋯*A*	*D*—H⋯*A*
N7—H2*N*7⋯O4^i^	0.89 (2)	2.15 (2)	3.0152 (19)	163.5 (18)
N7—H1*N*7⋯N4^ii^	0.89 (2)	2.43 (2)	3.1019 (17)	133.1 (17)
N7—H1*N*7⋯N5^ii^	0.89 (2)	2.45 (2)	3.0166 (16)	122.5 (16)
C3—H3*A*⋯O5^iii^	0.93	2.57	3.345 (3)	141
C14—H14*A*⋯O3^iv^	0.97	2.53	3.4443 (18)	157

Symmetry codes: (i) 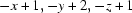; (ii) 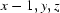; (iii) 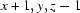; (iv) 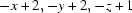.

**Fig. 1.**
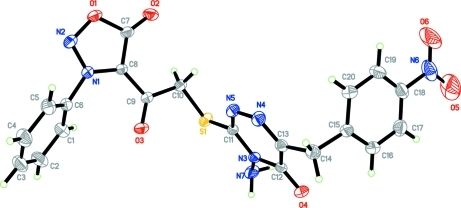
The molecular structure of the title compound, showing 30% probability displacement ellipsoids and the atom-numbering scheme. Hydrogen atoms are shown as spheres of arbitrary radius.

**Fig. 2.**
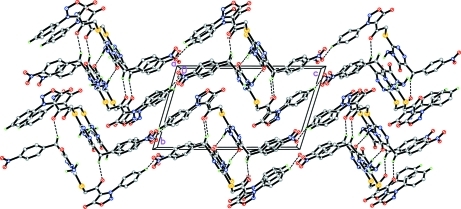
The crystal packing of (I) viewed along the a axis. Dashed lines indicate hydrogen bonds. H atoms not involved in the hydrogen bond interactions have been omitted for clarity.

## supplementary crystallographic information

### Comment

Sydnones are mesoionic heterocyclic aromatic compounds. The study of sydnones still remains a field of interest because of their electronic structures and also because of the varied types of biological activities displayed by some of them (Rai *et al.*, 2008). Recently sydnone derivatives were found to exhibit promising antimicrobial properties (Kalluraya *et al.*, 2008). Since their discovery, sydnones have shown diverse biological activities and it is thought that the meso-ionic nature of the sydnone ring promotes significant interactions with biological systems. Because of wide variety of properties displayed by sydnones ,we were prompted to synthesize a new S-substituted triazinones containing a sydnone  ring.

Photochemical bromination of 3-aryl-4-acetylsydnone afforded 3-aryl-4 bromoacetylsydnones.  Condensation of 4-amino-6-(p-nitrobenzyl)-3-sulfanyl-1,2,4-triazin-5(4H)-one with 3-phenyl-4-bromoacetylsydnones  yielded S-substituted triazinone derivatives (Jyothi *et al.*, 2008).

All parameters in (I), Fig. 1, are within normal ranges and comparable with the related structure (Fun *et al.*, 2011). The dihedral angle between oxadiazole (C7/C8/N1/N2/O1) and  triazine (C11/N3/C12/C13/N4/N5) groups is 86.94 (7)°. The oxadiazole and triazine groups make dihedral angles of 52.96 (8) and 83.08 (7)° with the C1–C6 phneyl ring and 9.51 (8) and 82.08 (7)° with the C15–C20 benzene ring, respectively.

In the crystal structure, the N7—H2N7···O4^i^, N7—H1N7···N4^ii^, N7—H1N7···N5^ii^, C3—H3A···O5^iii^ and C14—H14A···O3^iv^ intermolecular interactions (Table 1) link the molecules into two-dimensional sheets parallel to the *ac*-plane (Fig. 2).

### Experimental

To a solution of 4-bromoacetyl-3-phenylsydnone (0.01mol) and 4-amino-6-(p-nitrobenzyl)-3-sulfanyl-1,2,4-triazin-5(4H)-one (0.01mol) in ethanol, a catalytic amount of anhydrous sodium acetate was added. The solution was stirred at room temperature for 2-3 hours. The solid product that separated out was filtered and dried.  It was then recrystallized from ethanol. Crystals suitable for X-ray analysis were obtained from 1:2 mixtures of DMF and ethanol by slow evaporation.

### Refinement

N-bound H atoms were located in a difference Fourier map and were refined freely. The remaining H atoms were positioned geometrically and refined using a riding model, with C—H = 0.93 or 0.97 Å, and with *U*_iso_(H) = 1.2*U*_eq_(C).

### Crystal data

**Table:**

C_20_H_15_N_7_O_6_S	*Z* = 2
*M**_r_* = 481.45	*F*(000) = 496
Triclinic, *P*1	*D*_x_ = 1.510 Mg m^−^^3^
Hall symbol: -P 1	Mo *K*α radiation, λ = 0.71073 Å
*a* = 6.4071 (1) Å	Cell parameters from 5430 reflections
*b* = 10.1629 (2) Å	θ = 3.7–30.1°
*c* = 17.1521 (3) Å	µ = 0.21 mm^−^^1^
α = 106.372 (1)°	*T* = 297 K
β = 92.400 (1)°	Block, yellow
γ = 97.551 (1)°	0.51 × 0.34 × 0.17 mm
*V* = 1058.61 (3) Å^3^	

### Data collection

**Table:**

Bruker SMART APEXII CCD area-detector diffractometer	7793 independent reflections
Radiation source: fine-focus sealed tube	5330 reflections with *I* > 2σ(*I*)
graphite	*R*_int_ = 0.030
φ and ω scans	θ_max_ = 32.8°, θ_min_ = 2.1°
Absorption correction: multi-scan (*SADABS*; Bruker, 2009)	*h* = −9→9
*T*_min_ = 0.900, *T*_max_ = 0.965	*k* = −15→15
21821 measured reflections	*l* = −26→26

### Refinement

**Table:**

Refinement on *F*^2^	Primary atom site location: structure-invariant direct methods
Least-squares matrix: full	Secondary atom site location: difference Fourier map
*R*[*F*^2^ > 2σ(*F*^2^)] = 0.047	Hydrogen site location: inferred from neighbouring sites
*wR*(*F*^2^) = 0.131	H atoms treated by a mixture of independent and constrained refinement
*S* = 1.04	*w* = 1/[σ^2^(*F*_o_^2^) + (0.0615*P*)^2^ + 0.084*P*] where *P* = (*F*_o_^2^ + 2*F*_c_^2^)/3
7793 reflections	(Δ/σ)_max_ < 0.001
315 parameters	Δρ_max_ = 0.25 e Å^−^^3^
0 restraints	Δρ_min_ = −0.23 e Å^−^^3^

### Special details

**Table:**

Geometry. All esds (except the esd in the dihedral angle between two l.s. planes) are estimated using the full covariance matrix. The cell esds are taken into account individually in the estimation of esds in distances, angles and torsion angles; correlations between esds in cell parameters are only used when they are defined by crystal symmetry. An approximate (isotropic) treatment of cell esds is used for estimating esds involving l.s. planes.
Refinement. Refinement of F^2^ against ALL reflections. The weighted R-factor wR and goodness of fit S are based on F^2^, conventional R-factors R are based on F, with F set to zero for negative F^2^. The threshold expression of F^2^ > 2sigma(F^2^) is used only for calculating R-factors(gt) etc. and is not relevant to the choice of reflections for refinement. R-factors based on F^2^ are statistically about twice as large as those based on F, and R- factors based on ALL data will be even larger.

### Fractional atomic coordinates and isotropic or equivalent isotropic displacement parameters (Å^2^)

**Table:**

	*x*	*y*	*z*	*U*_iso_*/*U*_eq_	
S1	0.76158 (5)	0.56912 (4)	0.39458 (2)	0.04815 (10)	
N1	1.40303 (17)	0.45428 (11)	0.21692 (6)	0.0388 (2)	
N2	1.5454 (2)	0.37194 (13)	0.20533 (8)	0.0525 (3)	
N3	0.73930 (15)	0.81262 (11)	0.49757 (6)	0.0362 (2)	
N4	1.14452 (17)	0.85392 (13)	0.56156 (7)	0.0460 (3)	
N5	1.06998 (16)	0.73678 (12)	0.49822 (7)	0.0430 (2)	
N6	0.8522 (4)	0.87248 (18)	0.93514 (10)	0.0851 (6)	
N7	0.52842 (19)	0.77429 (15)	0.46377 (9)	0.0531 (3)	
O1	1.51411 (17)	0.29797 (10)	0.26042 (7)	0.0531 (3)	
O2	1.29331 (18)	0.28459 (11)	0.35900 (7)	0.0565 (3)	
O3	1.07909 (19)	0.62369 (11)	0.28170 (7)	0.0604 (3)	
O4	0.68160 (17)	1.01247 (11)	0.58890 (6)	0.0536 (3)	
O5	0.6980 (4)	0.9118 (2)	0.96790 (12)	0.1335 (8)	
O6	0.9522 (4)	0.7945 (2)	0.95653 (11)	0.1279 (8)	
C1	1.2266 (3)	0.54038 (16)	0.11647 (9)	0.0520 (3)	
H1A	1.1003	0.4875	0.1210	0.062*	
C2	1.2384 (3)	0.61847 (18)	0.06204 (10)	0.0643 (4)	
H2A	1.1181	0.6198	0.0302	0.077*	
C3	1.4275 (3)	0.69420 (17)	0.05489 (10)	0.0653 (5)	
H3A	1.4347	0.7444	0.0171	0.078*	
C4	1.6052 (3)	0.69646 (18)	0.10286 (11)	0.0660 (4)	
H4A	1.7315	0.7491	0.0980	0.079*	
C5	1.5974 (3)	0.62030 (16)	0.15882 (10)	0.0541 (3)	
H5A	1.7166	0.6213	0.1919	0.065*	
C6	1.4068 (2)	0.54316 (13)	0.16354 (7)	0.0412 (3)	
C7	1.3425 (2)	0.33732 (13)	0.30715 (8)	0.0411 (3)	
C8	1.27298 (19)	0.44150 (12)	0.27599 (7)	0.0352 (2)	
C9	1.1124 (2)	0.52770 (12)	0.30653 (7)	0.0382 (2)	
C10	0.9904 (2)	0.48508 (14)	0.37157 (8)	0.0438 (3)	
H10A	1.0834	0.5066	0.4210	0.053*	
H10B	0.9464	0.3855	0.3535	0.053*	
C11	0.87411 (17)	0.71923 (13)	0.47012 (7)	0.0347 (2)	
C12	0.8018 (2)	0.93081 (13)	0.56189 (7)	0.0380 (2)	
C13	1.0230 (2)	0.94417 (13)	0.59158 (7)	0.0392 (3)	
C14	1.1084 (2)	1.06316 (14)	0.66524 (8)	0.0484 (3)	
H14A	1.0497	1.1456	0.6635	0.058*	
H14B	1.2609	1.0831	0.6662	0.058*	
C15	1.0474 (2)	1.02257 (13)	0.74039 (8)	0.0418 (3)	
C16	0.8680 (3)	1.05963 (15)	0.77747 (9)	0.0516 (3)	
H16A	0.7898	1.1176	0.7592	0.062*	
C17	0.8036 (3)	1.01111 (17)	0.84163 (10)	0.0592 (4)	
H17A	0.6821	1.0350	0.8663	0.071*	
C18	0.9234 (3)	0.92666 (15)	0.86812 (9)	0.0575 (4)	
C19	1.1038 (3)	0.89030 (17)	0.83385 (10)	0.0652 (5)	
H19A	1.1834	0.8342	0.8533	0.078*	
C20	1.1651 (3)	0.93889 (16)	0.76961 (9)	0.0559 (4)	
H20A	1.2875	0.9151	0.7455	0.067*	
H2N7	0.492 (3)	0.848 (2)	0.4511 (12)	0.084 (6)*	
H1N7	0.456 (3)	0.764 (2)	0.5051 (13)	0.077 (6)*	

### Atomic displacement parameters (Å^2^)

**Table:**

	*U*^11^	*U*^22^	*U*^33^	*U*^12^	*U*^13^	*U*^23^
S1	0.03577 (16)	0.0519 (2)	0.0551 (2)	0.01356 (13)	0.00911 (13)	0.00898 (15)
N1	0.0436 (5)	0.0376 (5)	0.0387 (5)	0.0140 (4)	0.0093 (4)	0.0123 (4)
N2	0.0569 (7)	0.0522 (7)	0.0586 (7)	0.0271 (5)	0.0224 (6)	0.0211 (6)
N3	0.0302 (4)	0.0467 (5)	0.0407 (5)	0.0173 (4)	0.0105 (4)	0.0210 (4)
N4	0.0339 (5)	0.0572 (7)	0.0483 (6)	0.0106 (5)	0.0098 (4)	0.0150 (5)
N5	0.0310 (5)	0.0536 (6)	0.0477 (6)	0.0155 (4)	0.0102 (4)	0.0147 (5)
N6	0.1383 (18)	0.0596 (9)	0.0504 (8)	−0.0176 (10)	0.0025 (10)	0.0201 (7)
N7	0.0326 (5)	0.0653 (8)	0.0628 (8)	0.0238 (5)	0.0036 (5)	0.0135 (6)
O1	0.0586 (6)	0.0485 (5)	0.0657 (6)	0.0301 (5)	0.0198 (5)	0.0259 (5)
O2	0.0658 (7)	0.0529 (6)	0.0680 (6)	0.0225 (5)	0.0167 (5)	0.0374 (5)
O3	0.0766 (7)	0.0571 (6)	0.0694 (7)	0.0392 (6)	0.0293 (6)	0.0371 (5)
O4	0.0598 (6)	0.0541 (6)	0.0554 (6)	0.0336 (5)	0.0097 (5)	0.0177 (5)
O5	0.181 (2)	0.1328 (17)	0.1005 (13)	0.0027 (15)	0.0624 (14)	0.0588 (12)
O6	0.212 (2)	0.1014 (12)	0.0919 (11)	0.0126 (13)	0.0041 (13)	0.0687 (11)
C1	0.0579 (8)	0.0514 (8)	0.0478 (7)	0.0030 (6)	−0.0001 (6)	0.0193 (6)
C2	0.0845 (12)	0.0638 (10)	0.0494 (8)	0.0089 (9)	−0.0052 (8)	0.0264 (7)
C3	0.0971 (14)	0.0556 (9)	0.0515 (8)	0.0105 (9)	0.0159 (9)	0.0280 (7)
C4	0.0753 (11)	0.0606 (10)	0.0676 (10)	0.0017 (8)	0.0239 (9)	0.0287 (8)
C5	0.0531 (8)	0.0571 (8)	0.0553 (8)	0.0070 (7)	0.0109 (6)	0.0209 (7)
C6	0.0510 (7)	0.0393 (6)	0.0364 (6)	0.0113 (5)	0.0102 (5)	0.0131 (5)
C7	0.0440 (6)	0.0350 (6)	0.0491 (7)	0.0139 (5)	0.0080 (5)	0.0157 (5)
C8	0.0373 (6)	0.0334 (5)	0.0390 (6)	0.0113 (4)	0.0073 (4)	0.0139 (4)
C9	0.0417 (6)	0.0365 (6)	0.0401 (6)	0.0141 (5)	0.0074 (5)	0.0126 (5)
C10	0.0462 (7)	0.0436 (7)	0.0486 (7)	0.0187 (5)	0.0152 (5)	0.0178 (5)
C11	0.0305 (5)	0.0442 (6)	0.0386 (5)	0.0149 (4)	0.0137 (4)	0.0211 (5)
C12	0.0436 (6)	0.0423 (6)	0.0388 (6)	0.0177 (5)	0.0119 (5)	0.0230 (5)
C13	0.0400 (6)	0.0438 (6)	0.0413 (6)	0.0083 (5)	0.0116 (5)	0.0221 (5)
C14	0.0534 (8)	0.0445 (7)	0.0499 (7)	0.0040 (6)	0.0077 (6)	0.0190 (6)
C15	0.0499 (7)	0.0350 (6)	0.0403 (6)	0.0085 (5)	0.0014 (5)	0.0102 (5)
C16	0.0597 (9)	0.0499 (8)	0.0527 (8)	0.0211 (6)	0.0097 (6)	0.0205 (6)
C17	0.0697 (10)	0.0567 (9)	0.0513 (8)	0.0109 (7)	0.0181 (7)	0.0132 (7)
C18	0.0912 (12)	0.0417 (7)	0.0373 (6)	−0.0004 (7)	0.0020 (7)	0.0130 (5)
C19	0.0953 (13)	0.0524 (8)	0.0544 (9)	0.0241 (9)	−0.0070 (9)	0.0219 (7)
C20	0.0632 (9)	0.0554 (8)	0.0547 (8)	0.0252 (7)	0.0043 (7)	0.0177 (7)

### Geometric parameters (Å, °)

**Table:**

S1—C11	1.7448 (13)	C3—C4	1.370 (3)
S1—C10	1.7951 (13)	C3—H3A	0.9300
N1—N2	1.3013 (15)	C4—C5	1.391 (2)
N1—C8	1.3616 (15)	C4—H4A	0.9300
N1—C6	1.4550 (16)	C5—C6	1.380 (2)
N2—O1	1.3693 (15)	C5—H5A	0.9300
N3—C11	1.3660 (14)	C7—C8	1.4242 (16)
N3—C12	1.3820 (17)	C8—C9	1.4572 (16)
N3—N7	1.4079 (15)	C9—C10	1.5165 (17)
N4—C13	1.2914 (17)	C10—H10A	0.9700
N4—N5	1.3797 (16)	C10—H10B	0.9700
N5—C11	1.2966 (15)	C12—C13	1.4615 (18)
N6—O6	1.205 (3)	C13—C14	1.5049 (19)
N6—O5	1.214 (3)	C14—C15	1.5112 (18)
N6—C18	1.472 (2)	C14—H14A	0.9700
N7—H2N7	0.90 (2)	C14—H14B	0.9700
N7—H1N7	0.89 (2)	C15—C16	1.381 (2)
O1—C7	1.4212 (16)	C15—C20	1.3856 (19)
O2—C7	1.1937 (16)	C16—C17	1.384 (2)
O3—C9	1.2083 (15)	C16—H16A	0.9300
O4—C12	1.2156 (15)	C17—C18	1.377 (2)
C1—C6	1.373 (2)	C17—H17A	0.9300
C1—C2	1.384 (2)	C18—C19	1.366 (3)
C1—H1A	0.9300	C19—C20	1.380 (2)
C2—C3	1.377 (3)	C19—H19A	0.9300
C2—H2A	0.9300	C20—H20A	0.9300
			
C11—S1—C10	100.23 (6)	O3—C9—C10	123.28 (11)
N2—N1—C8	114.78 (10)	C8—C9—C10	113.88 (10)
N2—N1—C6	114.59 (10)	C9—C10—S1	113.41 (9)
C8—N1—C6	130.62 (10)	C9—C10—H10A	108.9
N1—N2—O1	105.29 (10)	S1—C10—H10A	108.9
C11—N3—C12	121.18 (10)	C9—C10—H10B	108.9
C11—N3—N7	116.60 (11)	S1—C10—H10B	108.9
C12—N3—N7	121.80 (10)	H10A—C10—H10B	107.7
C13—N4—N5	120.91 (11)	N5—C11—N3	123.75 (12)
C11—N5—N4	118.18 (11)	N5—C11—S1	121.37 (9)
O6—N6—O5	122.9 (2)	N3—C11—S1	114.86 (9)
O6—N6—C18	118.8 (2)	O4—C12—N3	122.00 (12)
O5—N6—C18	118.2 (2)	O4—C12—C13	125.50 (12)
N3—N7—H2N7	106.2 (14)	N3—C12—C13	112.50 (10)
N3—N7—H1N7	104.2 (14)	N4—C13—C12	123.40 (12)
H2N7—N7—H1N7	107.5 (19)	N4—C13—C14	118.21 (12)
N2—O1—C7	110.84 (9)	C12—C13—C14	118.20 (11)
C6—C1—C2	118.12 (15)	C13—C14—C15	108.09 (10)
C6—C1—H1A	120.9	C13—C14—H14A	110.1
C2—C1—H1A	120.9	C15—C14—H14A	110.1
C3—C2—C1	120.27 (16)	C13—C14—H14B	110.1
C3—C2—H2A	119.9	C15—C14—H14B	110.1
C1—C2—H2A	119.9	H14A—C14—H14B	108.4
C4—C3—C2	120.72 (14)	C16—C15—C20	119.09 (13)
C4—C3—H3A	119.6	C16—C15—C14	121.16 (12)
C2—C3—H3A	119.6	C20—C15—C14	119.60 (13)
C3—C4—C5	120.24 (16)	C15—C16—C17	120.48 (14)
C3—C4—H4A	119.9	C15—C16—H16A	119.8
C5—C4—H4A	119.9	C17—C16—H16A	119.8
C6—C5—C4	117.79 (16)	C18—C17—C16	118.61 (16)
C6—C5—H5A	121.1	C18—C17—H17A	120.7
C4—C5—H5A	121.1	C16—C17—H17A	120.7
C1—C6—C5	122.84 (13)	C19—C18—C17	122.36 (14)
C1—C6—N1	119.32 (12)	C19—C18—N6	119.06 (17)
C5—C6—N1	117.71 (13)	C17—C18—N6	118.58 (19)
O2—C7—O1	120.32 (11)	C18—C19—C20	118.29 (15)
O2—C7—C8	136.17 (13)	C18—C19—H19A	120.9
O1—C7—C8	103.50 (10)	C20—C19—H19A	120.9
N1—C8—C7	105.59 (10)	C19—C20—C15	121.14 (16)
N1—C8—C9	126.62 (10)	C19—C20—H20A	119.4
C7—C8—C9	127.47 (11)	C15—C20—H20A	119.4
O3—C9—C8	122.84 (12)		
			
C8—N1—N2—O1	−0.65 (15)	C12—N3—C11—N5	−3.89 (17)
C6—N1—N2—O1	−179.61 (10)	N7—N3—C11—N5	−176.56 (12)
C13—N4—N5—C11	−0.56 (18)	C12—N3—C11—S1	174.64 (8)
N1—N2—O1—C7	0.50 (15)	N7—N3—C11—S1	1.97 (14)
C6—C1—C2—C3	1.2 (3)	C10—S1—C11—N5	−5.61 (11)
C1—C2—C3—C4	−1.7 (3)	C10—S1—C11—N3	175.82 (8)
C2—C3—C4—C5	0.9 (3)	C11—N3—C12—O4	−177.39 (11)
C3—C4—C5—C6	0.4 (3)	N7—N3—C12—O4	−5.09 (18)
C2—C1—C6—C5	0.1 (2)	C11—N3—C12—C13	2.88 (15)
C2—C1—C6—N1	−175.51 (14)	N7—N3—C12—C13	175.17 (11)
C4—C5—C6—C1	−0.9 (2)	N5—N4—C13—C12	−0.10 (18)
C4—C5—C6—N1	174.80 (13)	N5—N4—C13—C14	174.79 (11)
N2—N1—C6—C1	124.32 (14)	O4—C12—C13—N4	179.22 (12)
C8—N1—C6—C1	−54.43 (19)	N3—C12—C13—N4	−1.05 (16)
N2—N1—C6—C5	−51.51 (17)	O4—C12—C13—C14	4.33 (18)
C8—N1—C6—C5	129.74 (15)	N3—C12—C13—C14	−175.94 (10)
N2—O1—C7—O2	−179.23 (13)	N4—C13—C14—C15	−92.98 (14)
N2—O1—C7—C8	−0.19 (14)	C12—C13—C14—C15	82.18 (14)
N2—N1—C8—C7	0.54 (15)	C13—C14—C15—C16	−94.98 (15)
C6—N1—C8—C7	179.29 (12)	C13—C14—C15—C20	80.63 (16)
N2—N1—C8—C9	174.43 (12)	C20—C15—C16—C17	−1.6 (2)
C6—N1—C8—C9	−6.8 (2)	C14—C15—C16—C17	174.06 (13)
O2—C7—C8—N1	178.62 (16)	C15—C16—C17—C18	0.7 (2)
O1—C7—C8—N1	−0.19 (13)	C16—C17—C18—C19	0.6 (2)
O2—C7—C8—C9	4.8 (3)	C16—C17—C18—N6	−178.61 (14)
O1—C7—C8—C9	−174.00 (12)	O6—N6—C18—C19	−1.9 (3)
N1—C8—C9—O3	−0.7 (2)	O5—N6—C18—C19	176.04 (19)
C7—C8—C9—O3	171.92 (13)	O6—N6—C18—C17	177.31 (17)
N1—C8—C9—C10	178.87 (12)	O5—N6—C18—C17	−4.7 (3)
C7—C8—C9—C10	−8.56 (19)	C17—C18—C19—C20	−1.0 (3)
O3—C9—C10—S1	10.82 (18)	N6—C18—C19—C20	178.23 (14)
C8—C9—C10—S1	−168.69 (9)	C18—C19—C20—C15	0.1 (2)
C11—S1—C10—C9	−88.06 (10)	C16—C15—C20—C19	1.2 (2)
N4—N5—C11—N3	2.54 (18)	C14—C15—C20—C19	−174.51 (14)
N4—N5—C11—S1	−175.89 (9)		

### Hydrogen-bond geometry (Å, °)

**Table:**

*D*—H···*A*	*D*—H	H···*A*	*D*···*A*	*D*—H···*A*
N7—H2N7···O4^i^	0.89 (2)	2.15 (2)	3.0152 (19)	163.5 (18)
N7—H1N7···N4^ii^	0.89 (2)	2.43 (2)	3.1019 (17)	133.1 (17)
N7—H1N7···N5^ii^	0.89 (2)	2.45 (2)	3.0166 (16)	122.5 (16)
C3—H3A···O5^iii^	0.93	2.57	3.345 (3)	141.
C14—H14A···O3^iv^	0.97	2.53	3.4443 (18)	157.

Symmetry codes: (i) −*x*+1, −*y*+2, −*z*+1; (ii) *x*−1, *y*, *z*; (iii) *x*+1, *y*, *z*−1; (iv) −*x*+2, −*y*+2, −*z*+1.

## References

[bb1] Bruker (2009). *APEX2*, *SAINT* and *SADABS* Bruker AXS Inc., Madison, Wisconsin, USA.

[bb2] Fun, H.-K., Quah, C. K., Nithinchandra, & Kalluraya, B. (2011). *Acta Cryst.* E**67**, o1004. 10.1107/S1600536811010798 PMC3099816 21754025

[bb3] Jyothi, C. H., Girisha, K. S., Adithya, A. & Kalluraya, B. (2008). *Eur. J. Med. Chem.* **43**, 2831–2834. 10.1016/j.ejmech.2008.02.003 18387710

[bb4] Kalluraya, B., Nayak, J., Adhikari, A., Sujith, K. V., Shetty, N. S. & Winter, M. (2008*a*). *Phosphorus Sulfur Silicon Relat. Elem.* **183**, 1870–1883.

[bb14] Kalluraya, B., Rao, J. N. & Sujith, K. V. (2008*b*). *Indian J. Heterocycl. Chem.* **17**, 359–362.

[bb5] Rai, N. S., Kalluraya, B., Lingappa, B., Shenoy, S. & Puranic, V. G. (2008). *Eur. J. Med. Chem.* **43**, 1715–1720. 10.1016/j.ejmech.2007.08.002 17923171

[bb6] Sheldrick, G. M. (2008). *Acta Cryst.* A**64**, 112–122. 10.1107/S0108767307043930 18156677

[bb7] Spek, A. L. (2009). *Acta Cryst.* D**65**, 148–155. 10.1107/S090744490804362X PMC2631630 19171970

